# Podocyte Metabolic Flexibility Under Stress Conditions: From Metabolic Adaptation to Maladaptive Responses

**DOI:** 10.3390/cells15191751

**Published:** 2026-09-26

**Authors:** Marine Thirion, Emmanuel Esteve, Vanessa Tagliatti, Anne-Emilie Declèves

**Affiliations:** 1 Laboratory of Metabolic and Molecular Biochemistry, Faculty of Medicine and Pharmacy, Research Institute for Health Sciences and Technology, University of Mons, 7000 Mons, Belgium; marine.thirion@umons.ac.be; 2 Laboratory of Human Biology and Toxicology, Faculty of Medicine and Pharmacy, Research Institute for Health Sciences and Technology, University of Mons, 7000 Mons, Belgium; vanessa.tagliatti@umons.ac.be; 3 French National Institute of Health and Medical Research (INSERM) CORAKID U155, Department of Nephrology, Assistance Publique-Hôpitaux de Paris (AP-HP), Tenon Hospital, Sorbonne University, 75020 Paris, France; emmanuel.esteve@aphp.fr

**Keywords:** podocyte, metabolism, glomerular disease, therapeutic target, mitochondria, chronic kidney disease

## Abstract

Podocytes are essential for glomerular function by maintaining the integrity of the glomerular filtration barrier. As terminally differentiated, post-mitotic cells, they are highly vulnerable to injury, which represents a central event in the development of chronic kidney disease. Although podocytes have been extensively studied to elucidate their structure and function, their metabolism and the role of metabolic dysregulation in podocyte injury remain incompletely understood. This review summarizes current knowledge of podocyte metabolism and its responses to diverse stressors. We discuss the basal metabolic profile of podocytes, which remains a matter of debate in the literature, with a focus on mitochondrial function. We further examine how stress conditions such as gluco- and lipotoxicity, vasoactive stress, mechanical stress, and immune dysregulation induce metabolic adaptations that become maladaptive upon sustained exposure. Metabolic reprogramming and its consequences, including cytoskeletal disorganization, are reviewed alongside emerging therapeutic strategies aimed at restoring podocyte metabolic homeostasis. A better understanding of podocyte metabolic flexibility may provide innovative strategies to preserve podocyte integrity and prevent the progression of chronic kidney disease.

## 1. Introduction

Podocytes are highly differentiated, post-mitotic epithelial cells that form the outer layer of the glomerular filtration barrier (GFB), together with the fenestrated endothelium and its glycocalyx and the glomerular basement membrane [1,2]. Their interdigitating foot processes (FPs) are connected by the slit diaphragm (SD), a specialized complex enriched in podocyte-specific proteins such as nephrin and podocin. The SD contributes to glomerular permselectivity as part of the integrated GFB, whose filtration properties depend on the combined structural and functional characteristics of its three layers and on the physicochemical properties of filtered molecules [3,4]. The highly specialized architecture of podocytes requires a continuous energy supply to preserve cytoskeletal organization, FP dynamics, and filtration barrier integrity, making these cells particularly vulnerable to disturbances in cellular bioenergetics.

The podocyte metabolic profile remains incompletely defined, but various metabolic alterations have been reported following injury, highlighting the central role of mitochondria in bioenergetics [5]. Podocyte injury can be triggered by a wide range of cellular stresses, resulting in cytoskeletal disorganization and foot process effacement and ultimately leading to podocyte death or detachment from the GBM [6,7]. Growing evidence highlights the crucial role of metabolic reprogramming, including the Warburg effect, in the development of chronic kidney disease (CKD), positioning the understanding of cellular metabolism at the center of potential therapeutic strategies [8].

The aim of this review is to synthesize current knowledge of the metabolic alterations that podocytes undergo in response to various cellular stresses. In doing so, we aim to improve our understanding of the underlying mechanisms involved in several podocyte-related diseases, such as focal segmental glomerulosclerosis (FSGS), diabetic nephropathy (DN), and minimal change disease (MCD) [9]. This review also aims to highlight the emerging importance of podocyte metabolism, which remains incompletely explored and, in some respects, controversial.

## 2. Podocyte Basal Metabolism

Because of their inability to proliferate and their critical role in maintaining the GFB through their interdigitating FPs, podocytes require a constant supply of energy to perform their functions. Cells primarily produce energy through two major pathways, namely glycolysis and oxidative phosphorylation (OXPHOS), which occur in the cytoplasm and mitochondria, respectively. However, discrepancies remain in the literature regarding the metabolic profile of podocytes and the relative contributions of these two major processes to adenosine triphosphate (ATP) generation. While certain studies support the idea that podocytes primarily rely on glycolysis, others highlight a crucial role for mitochondria and therefore OXPHOS in sustaining their function. Abe et al. [10] estimated that 77% of podocyte energy requirements are met through mitochondrial OXPHOS. In contrast, Brinkkoetter et al. [11] reported that podocyte metabolism predominantly relies on anaerobic glycolysis. In their study, podocyte-specific genetic deletion of key regulators of mitochondrial homeostasis, including peroxisome proliferator-activated receptor gamma coactivator 1-alpha (PGC-1α), dynamin-related protein 1 (Drp1), and mitochondrial transcription factor A (TFAM), did not result in any pathological phenotype [11]. A literature review reported that transmission electron microscopy studies showed mitochondria to be concentrated within the podocyte cell body and FPs, suggesting an important role in energy homeostasis [12]. However, these observations were obtained under physiological conditions, and podocyte metabolism may differ under pathological conditions. For instance, using podocyte-specific PHB2-deficient mice as a model of disrupted mitochondrial homeostasis, Özel et al. [13] demonstrated that deletion of OMA1, a key regulator of mitochondrial morphology, ameliorated the glomerular abnormalities observed in these animals. Mitochondrial dysfunction was also shown to sensitize podocytes to insulin, leading to overactivation of the mammalian target of rapamycin complex 1 (mTORC1) signaling pathway. Consistent with these results, OMA1 deficiency in isolated podocytes induced a metabolic shift toward glycolysis and enhanced glutamine anaplerosis upon insulin stimulation. By contrast, PHB2 deficiency in mice disrupted mitochondrial integrity and impaired respiratory function in podocytes exposed to insulin [14]. Collectively, these studies highlight the crucial role of mitochondrial regulators in modulating podocyte metabolism. Further supporting the importance of mitochondrial homeostasis in renal health, some rare hereditary mitochondrial disorders can present with renal manifestations [15].

Moreover, podocyte metabolism varies according to podocyte differentiation state. Indeed, conditionally immortalized podocyte models can be studied in distinct undifferentiated and differentiated states. During the transition between these two states, metabolic reprogramming occurs. Yuan et al. [16] demonstrated that podocyte differentiation is associated with increased glycolytic activity and enhanced mitochondrial metabolism, with differentiated podocytes displaying greater reliance on mitochondrial OXPHOS to meet their higher energy demands (Table 1).

In addition to the metabolic pathways involved, the nature of the main energy substrates used by podocytes also remains under debate. Renal cells utilize glucose and fatty acids to meet their energy demands. In proximal tubular cells, this is well established: they predominantly generate ATP through fatty acid oxidation (FAO) [17]. However, podocytes appear to exhibit greater metabolic flexibility. Several studies suggest that podocytes can utilize glucose, fatty acids, and alternative substrates. For example, under glucose-deprived conditions, podocytes have been shown to metabolize lactate, suggesting that lactate may contribute to maintaining energy homeostasis [18].

This remarkable metabolic flexibility may reflect the need for podocytes to rapidly adapt to changes in their environment, particularly under stress conditions.

## 3. Stressors and Associated Metabolic Response

### 3.1. Metabolic Stress

#### 3.1.1. Lipotoxicity

Lipid overload and lipotoxicity play a central role in podocyte injury, particularly in metabolic disorders such as obesity and diabetes [19,20]. In the context of obesity, abnormal lipid accumulation may disrupt podocyte homeostasis by altering cellular bioenergetics. Excess free fatty acids (FFAs), particularly saturated fatty acids (SFAs) such as palmitate (PA), contribute to oxidative stress, chronic inflammation, and insulin resistance, ultimately leading to podocyte apoptosis [21,22,23]. Several studies have shown that excessive accumulation of intracellular SFAs in podocytes leads to the production of toxic metabolites, such as diacylglycerol (DAG) and ceramides, which interfere with insulin signaling and promote inflammation [24]. Glomerular adenosine monophosphate (AMP)-activated protein kinase (AMPK) activity has also been shown to be reduced during the early stages of obesity-induced kidney disease in mice [25]. AMPK, a major cellular energy sensor, plays a central role in maintaining energy balance in response to nutritional and metabolic stress [26]. However, obesity has been shown to impair AMPK activation [27], thereby potentially limiting metabolic adaptation to lipid overload.

One of the most widely studied FFAs in in vitro models of obesity-associated lipotoxicity is PA, a saturated FFA abundant in Western diets. In podocytes, PA accumulation has been shown to induce mitochondrial dysfunction through excessive reactive oxygen species (ROS) production, as well as endoplasmic reticulum (ER) stress resulting from intrareticular calcium depletion and the accumulation of misfolded proteins. In this context, excessive oxidative stress impairs mitochondrial FAO, thereby promoting lipid droplet (LD) formation in the cytoplasm and increasing reliance on glycolysis even in the presence of oxygen [28]. This metabolic shift may represent a compensatory response aimed at protecting mitochondria from lipid overload. However, not all fatty acids exert similar effects. Oleate (OA), a monounsaturated FFA abundant in the Mediterranean diet, exerts protective effects in podocytes. Previous studies have demonstrated that OA counteracts PA-induced ER stress and significantly reduces podocyte cell death [29,30]. These observations indicate that lipotoxicity is not solely determined by FFA abundance but also by fatty acid composition and degree of saturation.

Growing evidence also indicates that sphingolipid metabolism is altered in obesity and diabetes. A study investigating the effects of human sera enriched in C16 ceramide on cultured human podocytes demonstrated modulation of mTORC1 signaling [31]. This regulation appeared to be lipoprotein-specific, as some lipoproteins significantly downregulated mTORC1 intermediates, whereas others upregulated them [31].

As lipid rafts are highly enriched within the slit diaphragm, cholesterol homeostasis is crucial for podocyte function, and excessive cholesterol accumulation can impair nephrin and podocin signaling. In high-fat diet (HFD)-fed mice, mitochondrial abnormalities have been observed in podocytes. More specifically, podocytes exhibited reduced expression of ATP-binding cassette transporter A1 (ABCA1), which mediates cholesterol and phospholipid efflux, leading to intracellular lipid accumulation. This resulted in mitochondrial cardiolipin accumulation, increased ROS generation, and ultimately podocyte injury [32]. Furthermore, lipidomic analyses performed in HFD-fed mice revealed that cholesteryl ester 20:4 reduced mitochondrial respiratory capacity and altered the expression of proteins involved in mitochondrial homeostasis, including Nrf2 and Drp1 [33,34].

Nephrotic syndromes are commonly associated with dyslipidemia, which may further contribute to podocyte metabolic stress. Remission following lipid apheresis has been reported; however, whether this effect results from lipid removal itself or from the concomitant elimination of harmful circulating factors remains unclear [35].

Altogether, lipid overload challenges podocyte metabolic homeostasis and can trigger both adaptive and maladaptive responses depending on lipid composition and the duration and magnitude of exposure. Beyond lipotoxicity, glucotoxicity represents another major metabolic challenge to podocyte homeostasis, particularly in diabetes and obesity.

#### 3.1.2. Glucotoxicity and Insulin Resistance

Glucose is a major substrate for ATP production in podocytes and is metabolized through both glycolysis and OXPHOS to meet cellular energy demands. Beyond bioenergetics, glucose is also metabolized through other pathways, such as the pentose phosphate pathway, which contributes to cellular redox homeostasis by maintaining the nicotinamide adenine dinucleotide phosphate (NADPH)/NADP^+^ ratio. Additional glucose-related pathways include the formation of advanced glycation end products (AGEs), the polyol pathway, the DAG-protein kinase C (PKC) pathway, and the hexosamine biosynthetic pathway (HBP).

Although glucose is essential for maintaining podocyte function, excessive glucose exposure associated with metabolic stress can lead to glucotoxicity, analogous to the lipotoxic effects induced by lipid overload. This condition is particularly relevant in diabetes, in which podocytes are chronically exposed to elevated glucose levels, promoting glucotoxicity and subsequent podocyte injury [36]. Excess glucose disrupts multiple cellular processes in podocytes, with increased oxidative stress and ROS generation representing central features of glucotoxicity. This occurs through multiple pathways, including the upregulation of NADPH oxidase 4 (Nox4) under diabetic conditions [37] and activation of the transforming growth factor-β (TGF-β)/Smad signaling pathway, a major driver of DN [38]. More recently, dual oxidase 2 (DUOX2) has been identified as another contributor, as its expression is increased in podocytes exposed to high glucose (HG) conditions, leading to enhanced ROS production, predominantly hydrogen peroxide (H_2_O_2_) [39]. Furthermore, glycogen synthase kinase-3β (GSK3β) is overactivated in type 2 diabetes and has been shown to alter Nrf2-mediated antioxidant responses, thereby enhancing oxidative stress and promoting podocyte injury and senescence [37]. In addition, GSK3β acts on insulin receptor substrate-1 (IRS-1), thereby contributing to insulin resistance and exacerbating metabolic alterations in podocytes.

Podocytes are insulin-sensitive cells in which insulin signaling regulates cytoskeletal organization, cellular bioenergetics, and survival through activation of the protein kinase B (Akt) pathway, thereby reducing apoptosis and regulating mTORC1 signaling. Altogether, these observations highlight the fundamental role of insulin in maintaining podocyte metabolic and functional homeostasis [14].

In parallel with metabolic dysregulation, glucotoxicity promotes chronic activation of inflammatory processes, primarily mediated through the nuclear factor-κB (NF-κB) signaling pathway. Activation of this pathway leads to the upregulation of regulated in development and deoxyribonucleic acid (DNA) damage response 1 (REDD1), a stress-responsive protein. Podocyte-specific deletion of REDD1 has been shown to attenuate inflammation under HG conditions, highlighting its involvement in chronic inflammation in DN [40]. Moreover, HG exposure promotes inflammation through activation of mTORC1, which induces the phosphorylation of signal transducer and activator of transcription 1 (STAT1), resulting in upregulation of complement factor B (CFB) expression [41].

Podocyte metabolic reprogramming occurs under HG conditions. Excess glucose disrupts the glycolysis/OXPHOS balance, favoring a metabolic switch toward glycolysis; however, the concomitant impairment of glycolytic flux suggests that this metabolic adaptation may become insufficient to maintain podocyte energy metabolism. In the glomeruli of db/db mice, ornithine catabolism is upregulated as a compensatory mechanism, leading to increased production of metabolites such as putrescine, which activates Ras homolog enriched in brain (Rheb), an upstream regulator of mTORC1 signaling. Pharmacological inhibition of ornithine decarboxylase 1 (ODC1) by difluoromethylornithine alleviates podocyte FP effacement in db/db mice [42]. Other glucose-derived metabolites have been identified as deleterious to podocyte function. Dihydroxyacetone phosphate (DHAP) accumulates when aldolase B is overexpressed, particularly under HG conditions, and induces mTORC1 activation. Both putrescine and DHAP promote oxidative stress and pyroptosis in podocytes [43]. As previously mentioned, glucose can also enter the HBP, with flux through this pathway increasing under hyperglycemic conditions. This results in increased levels of uridine diphosphate-N-acetylglucosamine (UDP-GlcNAc), a substrate for O-linked β-N-acetylglucosaminylation (O-GlcNAcylation) of proteins, thereby altering multiple cellular processes [44]. More recently, glycogen metabolism has also been shown to be dysregulated in hyperglycemia. Aberrant glycogen accumulation in podocytes disrupts intracellular signaling pathways and contributes to podocyte injury [45].

Finally, mitochondrial dysfunction driven by increased oxidative stress is a prominent feature of diabetic conditions and is closely associated with ER stress. Communication between mitochondria and the ER is mediated by mitochondria-associated ER membranes (MAMs). Under HG conditions, MAM integrity and abundance are reduced, highlighting the importance of mitochondria–ER crosstalk during glucotoxicity. Indeed, a study using cultured human podocytes demonstrated intracellular pyruvate accumulation resulting from mitochondrial dysfunction associated with acetylation of mitochondrial pyruvate carrier 2 (MPC2), a modification deleterious to podocyte function [46]. ER stress activates the unfolded protein response (UPR), which promotes cellular adaptation and survival by limiting apoptosis under hyperglycemic conditions. However, sustained ER stress contributes to cellular dysfunction.

Overall, the interplay between oxidative stress, inflammation, metabolic reprogramming, mitochondrial dysfunction, and ER stress can progressively shift podocyte responses from metabolic adaptation toward maladaptation, establishing a vicious cycle that exacerbates podocyte injury and ultimately contributes to progressive renal dysfunction [47,48]. Beyond gluco- and lipotoxicity, obesity and diabetes are characterized by glomerular hyperfiltration and elevated intraglomerular pressure, making podocytes highly susceptible to vasoactive stress, another major contributor to podocyte injury.

### 3.2. Vasoactive Stress

Beyond metabolic stress, podocytes are also exposed to vasoactive mediators that strongly influence their cellular metabolism and function. Under pathological conditions such as obesity, diabetes, and glomerular hyperfiltration, activation of the renin–angiotensin–aldosterone system (RAAS) and related signaling pathways occurs. This promotes oxidative stress, mitochondrial dysfunction, and activation of profibrotic pathways, ultimately leading to maladaptive responses in podocytes.

Luo et al. [49] demonstrated that immortalized human podocytes exposed to 10^−6^ M angiotensin II (Ang II) shifted their metabolism from glycolysis toward glycerol-3-phosphate (G3P) biosynthesis. Another study demonstrated that Ang II exposure in podocytes induces excessive ROS production, accompanied by decreased superoxide dismutase (SOD) activity, generation of mitochondrial superoxide anions, and upregulation of Nox4 through activation of Ang II receptor 1 (AT1R). This increase in oxidative stress ultimately triggers podocyte apoptosis and fibrosis through upregulation of TGF-β [50,51]. TGF-β signaling can promote oxidative stress in podocytes while also modulating their bioenergetics. TGF-β exposure leads to increased oxygen consumption rate (OCR) and extracellular acidification rate (ECAR), together with enhanced mitochondrial OXPHOS, reflecting substantial bioenergetic adaptation [52]. These metabolic changes are accompanied by mitochondrial dysfunction, including increased mitochondrial membrane potential, elevated ROS production, and upregulation of Nox4 messenger ribonucleic acid (mRNA) and protein levels [52]. Moreover, increased Nox4 expression contributes to podocyte apoptosis following TGF-β1 exposure [53]. Although most studies focus on TGF-β1, different TGF-β isoforms may exert distinct effects on podocyte metabolism. For example, Escasany et al. [54] demonstrated that TGF-β3 deficiency leads to podocyte FP effacement and albuminuria in heterozygous mice (*Tgfb3*+/−). Moreover, TGF-β3 deficiency is associated with lipid accumulation and dysregulation of lipid metabolism in mouse kidneys, supporting a potential renoprotective role for TGF-β3 in counteracting the harmful effects of TGF-β1 and TGF-β2.

In addition to Ang II, aldosterone can promote oxidative stress and podocyte injury through a distinct mechanism. Specifically, overactivation of the mineralocorticoid receptor (MR) in podocytes leads to increased ROS production, which in turn promotes ER stress. Activation of the UPR subsequently promotes C/EBP homologous protein (CHOP)-dependent apoptosis while also triggering protective autophagy as a compensatory response [55].

### 3.3. Mechanical Stress

Podocytes are continuously exposed to mechanical forces that increase under pathological conditions such as glomerular hypertension and hyperfiltration and contribute to podocyte injury and glomerulosclerosis [56]. Because maintenance of their complex architecture and dynamic cytoskeleton requires a continuous energy supply, increased mechanical stress may represent an important bioenergetic challenge for podocytes.

Endlich et al. [57] were the first to provide evidence that mechanical strain applied to cultured podocytes induced morphological alterations, actin cytoskeletal remodeling, and metabolic changes. Subsequent work further demonstrated that mechanical stress modulates podocyte metabolism, together with cell–matrix interactions and protein synthesis [58]. Mechanical stress also induces cytoskeletal remodeling, including loss of actin stress fibers and focal adhesions, illustrating the structural consequences of sustained mechanical load [59]. Mechanical stress may also reinforce vasoactive signaling through a feedback loop with RAAS activation, which itself induces metabolic alterations in podocytes, as described above. Mechanical stretching increases the expression of RAAS components, including AT1R [60], while Ang II signaling can promote redistribution of zonula occludens-1 (ZO-1) and nephrin downregulation, thereby further altering podocyte structural integrity [61]. Given their complex architecture and dynamic cytoskeleton, maintaining podocyte structural integrity under mechanical strain is likely to require substantial energy expenditure. Using a glomerulus-on-a-chip model, Doi et al. demonstrated that the application of mechanical forces alters podocyte sensitivity to puromycin aminonucleoside (PAN) and doxorubicin (DOX), two agents commonly used to induce experimental podocyte injury, and affects cellular metabolism [62]. Furthermore, another study reported that mechanical stress increases glucose uptake, especially under HG conditions [63], further supporting the importance of energy metabolism in podocyte adaptation to mechanical stress. Together, these observations support the concept that metabolic adaptation contributes to the podocyte response to increased mechanical load, whereas sustained mechanical stress may ultimately overwhelm these adaptive mechanisms and promote podocyte dysfunction.

### 3.4. Inflammation and Immune Dysregulation

Inflammation and immune dysregulation contribute to podocyte injury in several podocytopathies, including FSGS, MCD, and membranous nephropathy (MN). In primary FSGS and MCD, immune-mediated mechanisms and circulating permeability factors have been proposed to directly affect podocyte function [64,65,66]. Beyond their identification, a major challenge is to understand how these factors alter podocyte metabolism and mitochondrial homeostasis [67].

Among the proposed circulating permeability factors, soluble urokinase-type plasminogen activator receptor (suPAR) has been extensively studied, although its role in primary FSGS remains controversial [68,69]. Other proposed factors include anti-CD40 antibodies, CLCF1, and autoantibodies targeting nephrin [70]. Although their precise pathogenic roles remain incompletely understood, increasing evidence suggests that circulating factors can affect podocyte metabolic and mitochondrial homeostasis.

Metabolic and mitochondrial alterations appear particularly relevant to podocyte injury in FSGS. Mitochondrial injury has been identified as one of the earliest cellular alterations in FSGS, as demonstrated using an inducible podocyte-specific *Nphs2* knockout mouse model [71]. APOL1 risk variants, through interactions with suPAR and αvβ3 integrins, have been shown to increase lysosomal membrane permeability, leading to impaired autophagy and subsequent accumulation of damaged mitochondria. In parallel, human podocytes exposed to suPAR exhibit metabolic reprogramming toward glycolysis, supporting a central role for glycolytic pathways in podocyte injury. Notably, despite this shift toward glycolysis, a reduction in glycolytic ATP production has been reported in nephrotic syndrome following MCD or FSGS [72]. Furthermore, a recent in vitro study demonstrated that downregulation of transmembrane protein 30A (TMEM30A) in podocytes initiates injury through decreased expression of key glycolytic proteins [73]. Altogether, these observations suggest that an initially adaptive metabolic switch becomes maladaptive during chronic exposure to circulating permeability factors.

Circulating permeability factors can also induce alterations in podocyte lipid metabolism. Cultured human podocytes exposed to sera from patients with primary FSGS have shown marked LD accumulation, accompanied by increased expression of perilipin 2, an LD-associated structural protein and potential biomarker of FSGS [74]. However, these results could be explained by the lipid content of patients’ sera rather than by the effects of circulating factors themselves. Supporting this interpretation, another study characterized sera from patients with primary FSGS and examined their effects on cultured podocytes. Using mass spectrometry-based lipidomic analyses, researchers identified specific lipid profiles in FSGS sera that induced alterations in the podocyte metabolome upon exposure [75]. LD formation may initially represent a protective response to lipid overload; however, excessive lipid accumulation can become maladaptive, leading to loss of membrane integrity and FP dysregulation. The mechanisms by which circulating factors disrupt lipid metabolism remain poorly understood.

Considering the different types of stress and their associated metabolic responses, podocytes initially activate adaptive mechanisms to maintain cellular homeostasis. However, sustained exposure to stressors progressively promotes maladaptive metabolic reprogramming and mitochondrial dysfunction, ultimately resulting in podocyte dysfunction and loss (Figure 1).

## 4. Consequences of Disrupted Bioenergetics on Podocytes

### 4.1. Mitochondrial Dysfunction and Bioenergetic Collapse

Metabolic adaptations enable podocytes to maintain ATP production and cellular homeostasis during transient stress. However, when stress becomes sustained, compensatory metabolic reprogramming may become insufficient or maladaptive, resulting in mitochondrial dysfunction, impaired substrate utilization, oxidative stress, and ultimately bioenergetic failure. Because maintenance of the highly specialized podocyte architecture requires a continuous energy supply, disruption of ATP-generating pathways can directly compromise cellular structure and function.

Oxidative stress is a major driver of podocyte injury and is closely associated with mitochondrial dysfunction and impaired cellular bioenergetics [76]. Mitochondrial pathways affected during podocyte injury include mitochondrial dynamics and mitophagy, mitochondrial biogenesis, and mitochondrial protein quality control [77]. The consequences of mitochondrial and bioenergetic dysfunction have been extensively investigated in experimental podocyte injury models such as PAN- and DOX-induced nephropathy. Yu et al. [78] showed that PAN induced oxidative stress in podocytes through the Smad3–Nox4 pathway, resulting in multiple features of mitochondrial dysfunction, including decreased mitochondrial DNA (mtDNA) content, impaired respiratory complex activity, reduced mitochondrial membrane potential, and diminished antioxidant defenses. These alterations ultimately triggered metabolic and bioenergetic collapse, leading to apoptosis. Moreover, silencing Smad3 or Nox4 restored mitochondrial function and reduced podocyte apoptosis. Similarly, DOX induced mitochondrial dysfunction in podocytes through disruption of the PGC-1α–mitochondrial axis [79]. PGC-1α plays a key role in mitochondrial biogenesis; its downregulation leads to increased mitochondrial oxidative stress and enhanced podocyte apoptosis. DOX-induced injury is also associated with impaired autophagic function and reduced FAO, as shown by Matsuta et al. [80], further highlighting how disruption of mitochondrial quality control and substrate utilization can compromise podocyte bioenergetic homeostasis.

### 4.2. Cytoskeletal Remodeling and FP Effacement

A major structural consequence associated with sustained metabolic dysfunction is cytoskeletal remodeling accompanied by FP effacement. The actin cytoskeleton is fundamental to podocyte function, as it maintains the structure and integrity of the GFB and SD [81]. Because maintenance and dynamic remodeling of the podocyte cytoskeleton require a continuous energy supply, impaired bioenergetics and oxidative stress may compromise cytoskeletal organization. Across the various stressors described in the previous section, cytoskeletal remodeling emerges as an early manifestation of podocyte injury and is commonly observed in FSGS, DN, and MCD. SD-associated proteins, including nephrin, podocin, and ZO-1, are particularly vulnerable to sustained metabolic stress. For instance, exposure to HG conditions or TGF-β1 reduces nephrin expression in podocytes [82]. In addition, REDD1 promotes cytoskeletal remodeling under hyperglycemic conditions by inducing NF-κB-dependent transcription of TRPC6, leading to increased intracellular calcium. Activation of calcium-dependent signaling pathways drives actin cytoskeletal reorganization, ultimately resulting in FP effacement and proteinuria [83]. Supporting the link between oxidative stress and structural podocyte injury, quercetin attenuates FP effacement in rats with streptozotocin-induced DN by reducing oxidative stress and inhibiting the TGF-β1/Smad pathway [84].

### 4.3. Dedifferentiation, Partial EMT, and Cell-Cycle Dysregulation

Another phenotypic consequence associated with sustained podocyte stress is dedifferentiation, characterized by the progressive loss of podocyte-specific markers such as podocin and nephrin. This loss of podocyte identity may be accompanied by features of partial epithelial-to-mesenchymal transition (EMT), which contributes to phenotypic instability and may ultimately promote podocyte detachment from the GBM [85]. Although maintenance of the highly specialized podocyte phenotype is metabolically demanding, the extent to which bioenergetic failure directly drives podocyte dedifferentiation and partial EMT remains incompletely understood. Under sustained injury, podocytes may also aberrantly re-enter the cell cycle through altered expression of cell-cycle regulators. Because podocytes are terminally post-mitotic cells, they are unable to complete mitosis normally, resulting in defective chromosomal segregation, multinucleation, and ultimately mitotic catastrophe [86]. Rather than representing a primary metabolic response, these phenotypic alterations may therefore be viewed as downstream manifestations of sustained cellular stress occurring when adaptive mechanisms fail to preserve podocyte homeostasis.

### 4.4. Cell Death Pathways and Podocyte Loss

When metabolic adaptation becomes insufficient to maintain cellular homeostasis, persistent mitochondrial dysfunction, oxidative stress, and impaired energy metabolism can ultimately activate cell death pathways and promote podocyte loss. Several of the stressors described above promote podocyte apoptosis through both intrinsic (mitochondrial) and extrinsic (death receptor) pathways. The intrinsic pathway is particularly relevant to bioenergetic dysfunction because severe mitochondrial damage can promote cytochrome c release and subsequent caspase activation. HG exposure induces apoptosis in cultured podocytes through multiple mechanisms, including cytochrome P450 4A (CYP4A) upregulation, increased 20-hydroxyeicosatetraenoic acid (20-HETE) production, and Nox activation [87]. In addition to apoptosis, glucotoxicity has been associated with other cell death and stress pathways in podocytes, including pyroptosis, ferroptosis, and dysregulated autophagy [88]. Lipotoxicity similarly promotes mitochondria-dependent apoptosis through cytochrome c release and caspase activation [89]. In this context, berberine has been identified as a protective compound that attenuates podocyte apoptosis by inhibiting Drp1-mediated mitochondrial dysfunction. In db/db mice, berberine confers protection against gluco- and lipotoxicity-induced podocyte damage by reducing ROS production [90].

The ultimate consequences of podocyte death and loss are functional impairments that primarily manifest as proteinuria, in particular albuminuria, together with progressive deterioration of glomerular function. Given the limited regenerative capacity of podocytes, sustained podocyte depletion can promote glomerulosclerosis and progressive kidney dysfunction, particularly in diseases such as FSGS and DN [2,7].

Consequently, preserving podocyte integrity requires maintaining metabolic and mitochondrial homeostasis to support their specialized functions. As underscored throughout this review, podocyte metabolism plays a key role in cellular adaptation to stress, supporting the development of therapeutic strategies aimed at preventing maladaptive metabolic responses and restoring podocyte homeostasis (Figure 2).

## 5. Therapeutic Strategies Targeting Mitochondrial Homeostasis

Current therapeutic strategies for glomerular diseases mainly rely on RAAS blockade, immunosuppressive agents, and sodium–glucose cotransporter 2 (SGLT2) inhibitors. These treatments effectively reduce proteinuria, glomerular hypertension, and inflammation, thereby slowing CKD progression. However, their efficacy remains limited. Moreover, these approaches do not directly target podocyte metabolic disturbances or mitochondrial impairment. Consequently, an emerging therapeutic strategy is to target mitochondrial dysfunction, a prominent feature of podocyte injury, with the aim of restoring podocyte metabolic homeostasis. Potential therapeutic approaches include modulation of key metabolic and mitochondrial regulatory pathways, including AMPK, mTORC1, and sirtuins [91,92,93]. Sirtuins are important regulators of mitochondrial homeostasis, oxidative stress, and cellular metabolism [94]. In podocytes, sirtuin 1 (SIRT1) has been shown to exert protective effects by preserving mitochondrial function and limiting oxidative stress, whereas impaired SIRT1 signaling is associated with podocyte injury [95]. In parallel, decreased expression of components of the PTEN-induced kinase 1 (PINK1)-mediated mitophagy pathway has been reported in injured podocytes. Notably, this defect can be partially reversed with the mitochondria-targeted antioxidant MitoTEMPO [96]. Another emerging strategy involves the use of mesenchymal stromal cells to transfer healthy mitochondria to injured podocytes via M-Sec-dependent tunneling nanotubes. This mitochondrial transfer restores mitochondrial function, reduces apoptosis, and increases nephrin abundance in podocytes [97]. Mitochondrial dynamics also represent an innovative therapeutic target. Berberine, as previously discussed, inhibits excessive mitochondrial fission, thereby stabilizing mitochondrial networks [90]. Similarly, astragaloside II partially restores the expression of proteins involved in mitochondrial dynamics, such as mitofusin 2 (MFN2) and mitochondrial fission 1 protein (Fis1) [98]. Activation of mitochondrial G3P dehydrogenase (mGPDH) has been shown to enhance mitochondrial biogenesis and function in mouse models of glomerular injury [99]. Finally, elamipretide, a mitochondria-targeted peptide that stabilizes cardiolipin and limits its peroxidation, has been shown to ameliorate DN and preserve podocyte integrity, highlighting its potential as a therapeutic strategy targeting mitochondrial dysfunction [100]. Altogether, targeting mitochondrial homeostasis may attenuate podocyte injury and preserve glomerular function (Table 2).

In line with this approach, restoring redox homeostasis represents a promising strategy for treating podocyte injury through antioxidant therapies. This can be achieved by targeting the Nrf2–Keap1 signaling pathway, a central regulator of the cellular antioxidant response. Sulforaphane, a potent Nrf2 activator, has been shown to attenuate podocyte injury in DN through activation of the Nrf2/PINK1 pathway. In diabetic mouse models, sulforaphane treatment improved the albumin-to-creatinine ratio and prevented glomerular hypertrophy [101]. Similarly, quercetin, a natural flavonoid with antioxidant properties, protects against podocyte injury by reducing oxidative stress and inhibiting TGF-β1/Smad signaling [84]. In addition, probucol has been reported to attenuate oxidative stress by downregulating Nox2 expression and reducing thiobarbituric acid-reactive substances (TBARS) production in type 2 diabetes [102]. Selective targeting of specific Nox isoforms may represent a promising therapeutic strategy for podocyte injury.

Finally, as discussed above, alterations in lipid metabolism, particularly in the context of lipotoxicity-induced podocyte injury, have prompted the development of therapeutic strategies targeting lipid metabolism. Among potential targets, carnitine palmitoyltransferase 1A (CPT1A), the rate-limiting enzyme of FAO, has emerged as a key regulator of podocyte lipid metabolism. CPT1A overexpression has been shown to reduce albuminuria and glomerulosclerosis by promoting fatty acid utilization and reducing lipid accumulation and lipotoxicity in podocytes, highlighting CPT1A as a potential therapeutic target in podocyte injury [89]. In addition, A30, a pharmacological inducer of ABCA1, has been shown to ameliorate podocyte injury and DN by restoring ABCA1-dependent lipid homeostasis. A30 exposure reduces levels of docosahexaenoic acid-rich cardiolipin, thereby restoring mitochondrial lipid composition and improving the renal phenotype [100]. Enhancement of chaperone-mediated autophagy has also been reported to promote perilipin 2 degradation, thereby reducing intracellular lipid accumulation and attenuating hyperlipidemia-induced podocyte injury [103]. Finally, small molecules directly targeting LD structure and function are currently under development and may protect podocytes from cell death by inducing lipophagy [104]. Overall, a deeper understanding of podocyte metabolism may reveal novel therapeutic opportunities to preserve podocyte health and limit the progression of glomerular diseases.

## 6. Conclusions and Perspectives

Podocytes are essential for maintaining glomerular health by preserving GFB integrity through the SD and a highly organized actin cytoskeleton. As terminally differentiated, post-mitotic cells, podocytes are particularly vulnerable to a wide range of stressors, and their loss compromises glomerular function, leading to proteinuria and contributing to CKD development and progression.

Although no consensus has yet been reached regarding podocyte metabolism, accumulating evidence points to a central role for mitochondria in meeting the high energy demands of podocytes. Podocytes display remarkable metabolic flexibility, enabling rapid switching between metabolic pathways to adapt to environmental stressors. Nevertheless, beyond this adaptive phase, sustained metabolic changes become maladaptive, promoting podocyte injury, cell death, and detachment from the GBM.

Mitochondrial dysfunction emerges as a common pathological feature across diverse stress conditions, including gluco- and lipotoxicity, vasoactive stress, mechanical stress, and exposure to circulating permeability factors. In this context, oxidative stress represents a major driver of podocyte injury and is associated with dedifferentiation, maladaptive metabolic shifts, and cytoskeletal disorganization.

To better elucidate podocyte metabolic regulation, integrated approaches combining multi-omics technologies are required. In parallel, the development of more physiologically relevant models is essential, as conventional two-dimensional cultures have limitations in mimicking the complex microenvironment of the glomerulus. Advanced systems, such as glomerulus-on-a-chip platforms, represent promising approaches for modeling the complex glomerular microenvironment.

Targeting metabolic alterations therefore represents a promising therapeutic strategy to preserve podocyte health and limit the progression of glomerular disease. Emerging metabolism-based therapeutic strategies, together with the identification of innovative biomarkers, hold significant promise for improving patient stratification, monitoring disease progression, and ultimately improving the management of CKD.

## Figures and Tables

**Figure 1:**
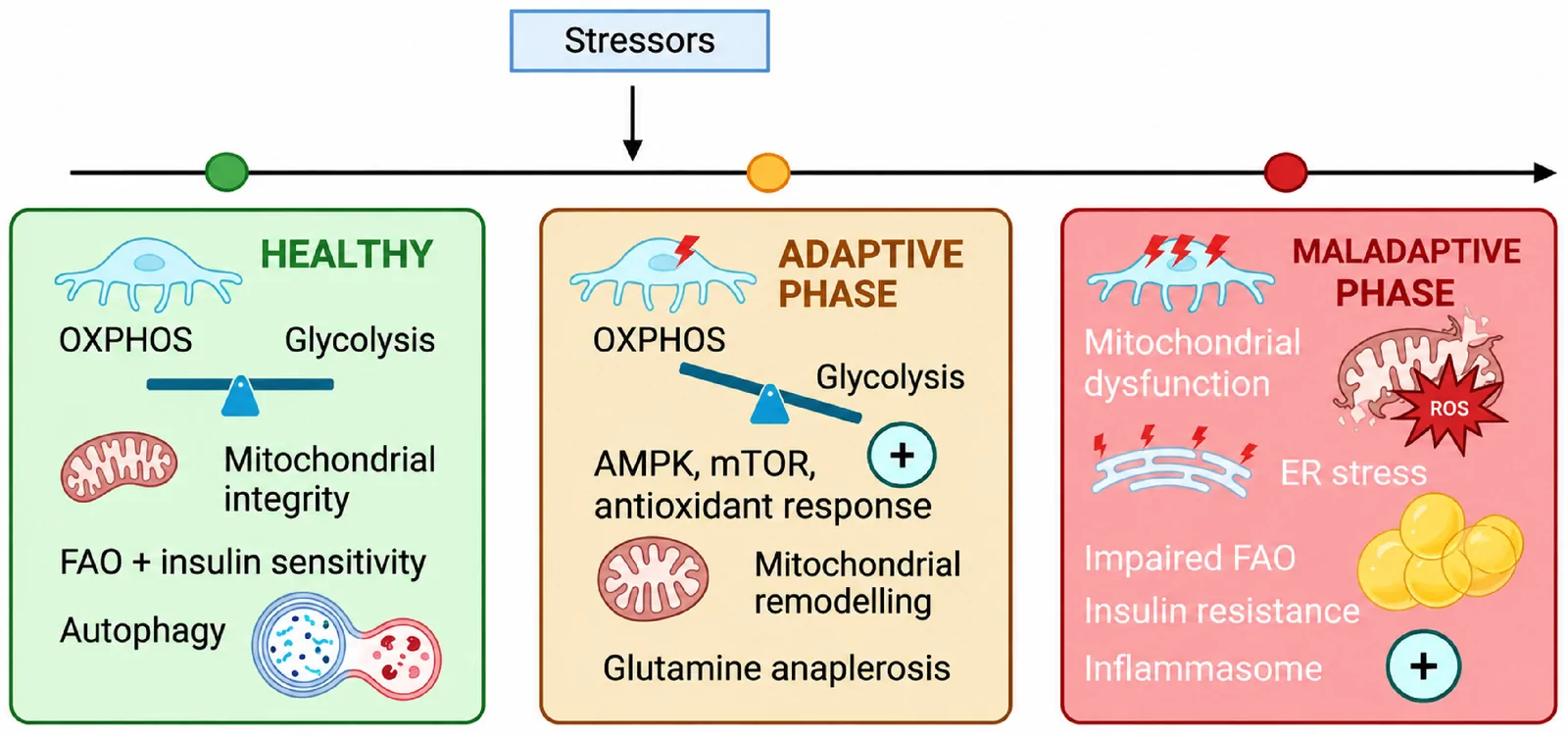
Metabolic reprogramming in podocytes following acute (adaptive phase) and chronic (maladaptive phase) exposure to various stressors. Abbreviations: OXPHOS: oxidative phosphorylation; FAO: fatty acid oxidation; AMPK: adenosine monophosphate-activated protein kinase; mTOR: mammalian target of rapamycin; ER: endoplasmic reticulum. + indicates upregulation or activation.

**Figure 2:**
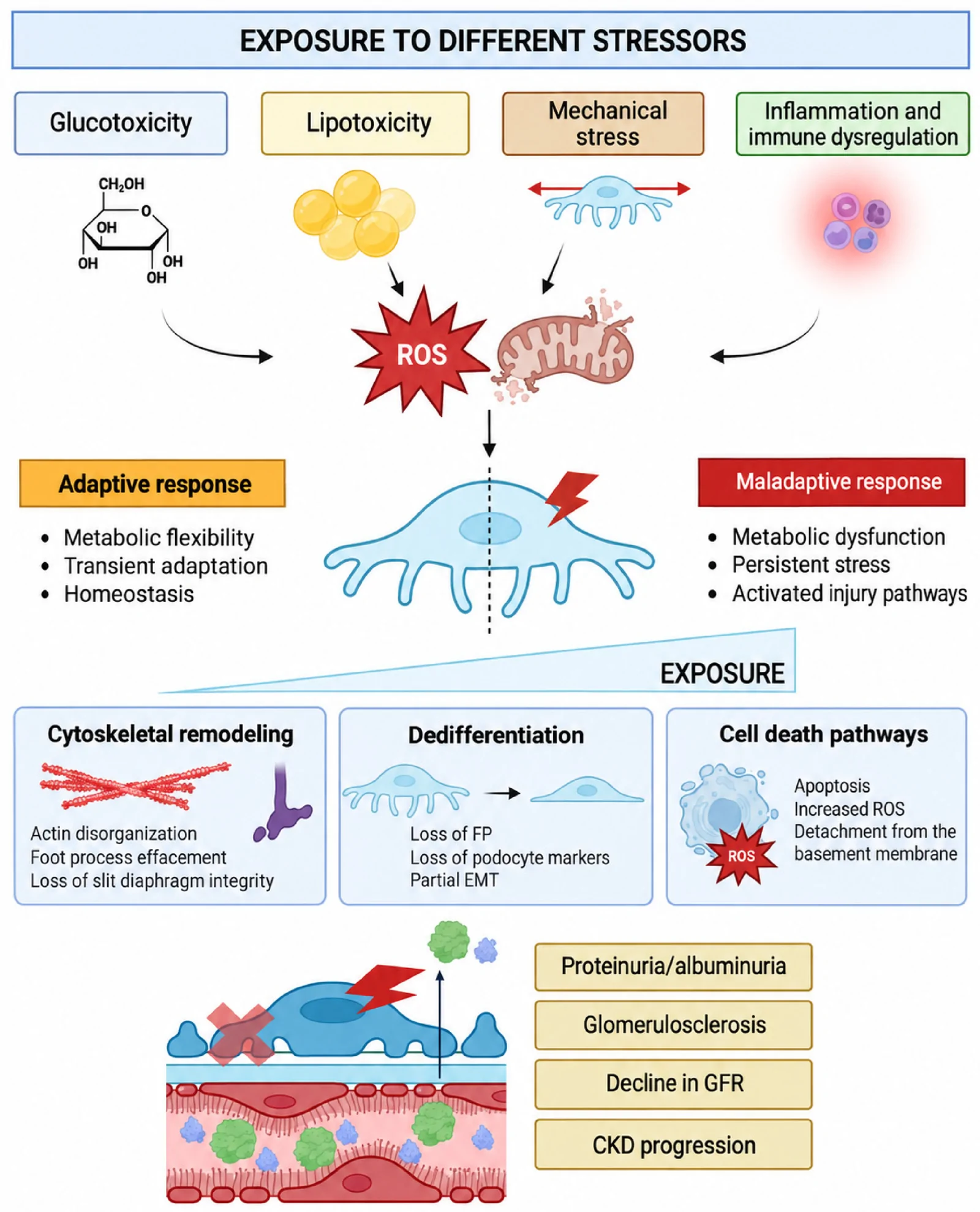
Podocyte exposure to different stressors and their consequences. Abbreviations: FP: foot process; EMT: epithelial-to-mesenchymal transition; ROS: reactive oxygen species; GFR: glomerular filtration rate; CKD: chronic kidney disease. The cross (x) indicates podocyte loss.

**Table 1:** Current evidence and controversies regarding basal podocyte metabolism. Abbreviations: OXPHOS: oxidative phosphorylation; Tfam: mitochondrial transcription factor A; Dnm1l: dynamin-related protein 1; Ppargc1a: peroxisome proliferator-activated receptor gamma coactivator 1-alpha; Phb2: prohibitin 2; Oma1: OMA1 zinc metallopeptidase; mTOR: mammalian target of rapamycin; Pkm2: pyruvate kinase M2.

Physiological/Pathological Condition	Model	Main Metabolic Pathway	Key Findings	Ref.
Physiological	Transformed mouse podocyte cell line, primary mouse podocytes	OXPHOS	Importance of mitochondria in energy production	[10]
Physiological Pathological	Podocyte-specific *Tfam* knockout mice, podocyte-specific *Dnm1l* knockout mice, podocyte-specific *Ppargc1a* knockout mice	Glycolysis	Importance of the glycolytic pathway in energy production Disruption of mitochondrial turnover did not impair podocyte function	[11]
Physiological (review)	Literature review on podocyte energetics	Metabolic flexibility between glycolysis and OXPHOS	Mitochondria are essential for maintaining cytoskeletal function Metabolic alterations contribute to podocyte injury	[12]
Pathological (mitochondrial dysfunction)	Podocyte-specific *Phb2* knockout mice, *Oma1* deletion, isolated podocytes	Glycolysis, glutamine anaplerosis, mTOR signaling, OXPHOS	Mitochondrial dysfunction sensitized podocytes to insulin and induced mTOR hyperactivation	[13]
Pathological (mitochondrial dysfunction)	Conditionally immortalized mouse podocytes with *Oma1* deletion or inducible PHB2 knockdown	Glycolysis, glutamine anaplerosis, OXPHOS, metabolic flexibility	Compensatory glycolytic shift in response to mitochondrial dysfunction	[14]
Physiological (podocyte differentiation)	Differentiated cultured mouse podocytes, podocyte-specific *Pkm2* knockout mice	Glycolysis, OXPHOS, PKM2-mediated metabolic reprogramming	Podocyte differentiation was associated with increased glycolysis and enhanced mitochondrial metabolism	[16]

**Table 2:** Innovative therapeutic strategies targeting mitochondrial dysfunction in podocyte injury. Abbreviations: ROS: reactive oxygen species; PINK1: PTEN-induced kinase 1; CKD: chronic kidney disease; TNF-α: tumor necrosis factor-α; DN: diabetic nephropathy; mGPDH: mitochondrial glycerol-3-phosphate dehydrogenase; HG: high glucose; FP: foot process; C-BSA: cationic bovine serum albumin.

Compound/Strategy Target Pathway	Mechanism	Podocyte Effect	Disease Model	Ref.
MitoTEMPO Mitochondrial oxidative stress, mitophagy	Mitochondria-targeted antioxidant reducing mitochondrial ROS and activating PINK1/Parkin-mediated mitophagy	Reduced podocyte injury, improved mitochondrial membrane potential, and decreased inflammasome activation	C-BSA-induced CKD rats and TNF-α-treated human podocytes	[96]
Mesenchymal stromal cells Mitochondrial dysfunction	Transfer of healthy mitochondria to injured podocytes through M-Sec-dependent tunneling nanotubes	Improved mitochondrial function, reduced apoptosis	Podocytes exposed to diabetes-related insults in vitro and experimental DN mice	[97]
Astragaloside II Mitochondrial dynamics, mitophagy, oxidative stress	Restoration of mitochondrial dynamics-related proteins and reduction in oxidative stress	Attenuated podocyte injury, reduced apoptosis, and improved mitochondrial morphology	Streptozotocin-induced diabetic rats	[98]
mGPDH restoration or activation mGPDH pathway	Enhancement of mitochondrial function and biogenesis, preservation of mitochondrial homeostasis, and reduction in oxidative stress	Reduced podocyte injury and apoptosis, improved mitochondrial function, and preserved slit diaphragm integrity	Diabetic kidney disease and Adriamycin nephropathy mouse models; HG-treated podocytes	[99]
Elamipretide Mitochondrial cardiolipin peroxidation	Stabilization of cardiolipin at the inner mitochondrial membrane and inhibition of cardiolipin peroxidation	Reduced podocyte loss and restored FP morphology	BTBR^ob/ob mice with established DKD and ABCA1-deficient podocyte models	[100]

## Data Availability

No new data were created or analyzed in this study. Data sharing is not applicable to this article.

## Abbreviations

The following abbreviations are used in this manuscript:

GFB	Glomerular filtration barrier
GBM	Glomerular basement membrane
FP	Foot process
SD	Slit diaphragm
CKD	Chronic kidney disease
FSGS	Focal segmental glomerulosclerosis
DN	Diabetic nephropathy
MCD	Minimal change disease
OXPHOS	Oxidative phosphorylation
ATP	Adenosine triphosphate
PGC-1α	Peroxisome proliferator-activated receptor gamma coactivator 1-alpha
Drp1	Dynamin-related protein 1
TFAM	Mitochondrial transcription factor A
mTORC1	Mammalian target of rapamycin complex 1
FAO	Fatty acid oxidation
FFAs	Free fatty acids
SFAs	Saturated fatty acids
PA	Palmitate
DAG	Diacylglycerol
LD	Lipid droplet
AMP	Adenosine monophosphate
AMPK	AMP-activated protein kinase
ROS	Reactive oxygen species
ER	Endoplasmic reticulum
OA	Oleate
ABCA1	ATP-binding cassette transporter A1
HFD	High-fat diet
Nrf2	Nuclear factor erythroid 2-related factor 2
NADPH	Nicotinamide adenine dinucleotide phosphate
AGEs	Advanced glycation end products
PKC	Protein kinase C
HBP	Hexosamine biosynthetic pathway
ESKD	End-stage kidney disease
Nox4	NADPH oxidase 4
HG	High glucose
TGF-β	Transforming growth factor-β
DUOX2	Dual oxidase 2
H_2_O_2_	Hydrogen peroxide
GSK3β	Glycogen synthase kinase-3β
IRS-1	Insulin receptor substrate-1
Akt	Protein kinase B
NF-κB	Nuclear factor-κB
DNA	Deoxyribonucleic acid
REDD1	Regulated in development and DNA damage response 1
STAT1	Signal transducer and activator of transcription 1
CFB	Complement factor B
Rheb	Ras homolog enriched in brain
ODC1	Ornithine decarboxylase 1
DHAP	Dihydroxyacetone phosphate
UDP-GlcNAc	UDP-N-acetylglucosamine
O-GlcNAcylation	O-linked β-N-acetylglucosaminylation
MAMs	Mitochondria-associated ER membranes
MPC2	Mitochondrial pyruvate carrier 2
UPR	Unfolded protein response
Ang II	Angiotensin II
G3P	Glycerol-3-phosphate
SOD	Superoxide dismutase
NADH	Nicotinamide adenine dinucleotide
AT1R	Ang II receptor type 1
OCR	Oxygen consumption rate
ECAR	Extracellular acidification rate
mRNA	Messenger ribonucleic acid
RAAS	Renin–angiotensin–aldosterone system
MR	Mineralocorticoid receptor
CHOP	C/EBP homologous protein
TRPC6	Transient receptor potential cation channel 6
ZO-1	Zonula occludens-1
PAN	Puromycin aminonucleoside
DOX	Doxorubicin
MN	Membranous nephropathy
suPAR	Soluble urokinase-type plasminogen activator receptor
TNF-α	Tumor necrosis factor-α
CLCF1	Cardiotrophin-like cytokine factor 1
TMEM30A	Transmembrane protein 30A
mtDNA	Mitochondrial DNA
CYP4A	Cytochrome P450 4A
20-HETE	20-hydroxyeicosatetraenoic acid
EMT	Epithelial-to-mesenchymal transition
SGLT2	Sodium-glucose cotransporter 2
MFN2	Mitofusin 2
mGPDH	Mitochondrial G3P dehydrogenase
TBARS	Thiobarbituric acid-reactive substances
SIRT1	Sirtuin 1
PINK1	PTEN-induced kinase 1
CPT1A	Carnitine palmitoyltransferase 1A
Fis1	Mitochondrial fission 1 protein
